# Reply to Poddighe

**DOI:** 10.1093/infdis/jix365

**Published:** 2017-09-06

**Authors:** Louise Stevenson, L-M Huang, Valérie Berlaimont, Nicolas Folschweiller

**Affiliations:** 1 GSK, Wavre, Belgium; 2 Department of Pediatrics, National Taiwan University Children’s Hospital, National Taiwan University College of Medicine, Taipei, Taiwan

To the Editor*—*We thank Poddighe for the critical review of our article related to the immunogenicity and safety of the AS04-adjuvanted human papillomavirus (HPV) type 16/18 (AS04-HPV-16/18) vaccine, given as 2-dose schedules and compared to a 3-dose schedule, and for his questions related to the safety of HPV vaccine.

The benefit/risk balance of the AS04-HPV16/18 vaccine is under constant evaluation by the Company through close monitoring of adverse event (AE) reports following vaccination allowing assessment of potential risk associated with vaccination. Various strategies are used, which include the long-term follow-up of cohorts vaccinated in clinical trials, pooling of clinical trials data, postmarketing surveillance of safety and clinical outcomes, and review of large health databases to enable signal detection [1]. As the AS04-HPV-16/18 vaccine is adjuvanted, the potential to develop autoimmune diseases is a theoretical concern. Consequently, the analysis of potentially immune-mediated diseases (pIMDs) is of particular interest.

A pooled analysis of the safety data from the GSK clinical trial development program (57 580 subjects and 96 704 vaccine doses) concluded that the incidence and distribution of AEs were similar among vaccine and control groups [2]. The rates of pIMD between groups were also similar, with no patterns in the specific syndrome reported or the time to onset. Similarly, an analysis of postlicensure safety data of >4 years of routine clinical practice identified no safety concerns from general AE reports, or from reports of pIMDs [3]. An observational cohort study in female subjects aged 9–25 years did not show evidence of an increased risk of autoimmune disease following vaccination with AS04-HPV-16/18 vaccine [4]. More recently, several analyses looking at specific AEs of potential interest after HPV vaccination implementation and conducted by independent institutions have been published and have come to similar conclusions [5–9].

In the study by Huang et al, all subjects received the AS04-HPV-16/18 vaccine. Medically significant conditions, defined as AEs prompting emergency room or physician visits with the exception of routine visits or common diseases, were to be reported throughout the study. Analysis of the 374 reported medically significant conditions did not reveal any event or cluster of events of potential concern. Serious AEs (SAEs) were reported by 72 participants, none of which were fatal. Appendicitis was the most frequently reported SAE, by 8 subjects. Most SAEs were reported only once per group. The pIMD case of systemic erythematosus lupus (SLE) described by Huang et al was assessed as causally related to vaccination by the investigator while the study was ongoing. The exact time to onset of the symptoms is unclear. The patient had right knee pain from an unspecified date and received a diagnosis of transient synovitis 6 months after first vaccination. A diagnosis of SLE was obtained almost 2 years after the initial vaccination. Retrospectively, the case did not fully meet criteria for SLE [10] and could not be confirmed on the basis of available information. Of interest, a recent study investigated the risk of SLE in vaccinated subjects (most of whom received non-HPV vaccines) and did not make conclusions regarding vaccine causality [11]. The second event of interest described by Huang et al, celiac disease, was classified as a nonserious event. The diagnosis was made 3 weeks after first vaccination; however, no confirmatory biopsy was performed, to our knowledge. The subject’s recorded medical history suggested preexisting manifestations of the disease. No further information was available, as the subject withdrew from the study shortly after receiving the first dose and did not wish to be recontacted.

In conclusion, the safety data generated in the present study were in line with the known safety profile of the vaccine as described in the current product information [12]. The full clinical study report is available from the GSK clinical trial register website (available at: https://www.gsk-clinicalstudyregister.com), under study ID 114700.

## Notes

***Acknowledgments.*** The authors would like to thank Business & Decision Life Sciences platform for editorial assistance and publication coordination, on behalf of GSK. Thibaud André (Business & Decision Life Sciences on behalf of GSK) coordinated the publication development and provided editorial support.

***Financial support.*** This work was supported by GlaxoSmithKline Biologicals SA.

***Potential conflicts of interest.*** N. F., V. B., and L. S. are employees of the GSK group of companies. N. F. and V. B. received restricted shares from the GSK group of companies. L.-M. H. received grants through his institution from the GSK group of companies and also received consultancy fees for participation in the HPV expert board and payment for educational presentation from the GSK group of companies. All authors have submitted the ICMJE Form for Disclosure of Potential Conflicts of Interest. Conflicts that the editors consider relevant to the content of the manuscript have been disclosed.
